# Oral Vaccination With Recombinant *Pichia pastoris* Expressing Iridovirus Major Capsid Protein Elicits Protective Immunity in Largemouth Bass (*Micropterus salmoides*)

**DOI:** 10.3389/fimmu.2022.852300

**Published:** 2022-03-04

**Authors:** Jia-Yun Yao, Cheng-Sai Zhang, Xue-Mei Yuan, Lei Huang, Da-Yan Hu, Zhe Yu, Wen-Lin Yin, Ling-Yun Lin, Xiao-Yi Pan, Gui-lian Yang, Chun-Feng Wang, Jin-Yu Shen, Hai-Qi Zhang

**Affiliations:** ^1^ Agriculture Ministry Key Laboratory of Healthy Freshwater Aquaculture, Key Laboratory of Fish Health and Nutrition of Zhejiang Province, Zhejiang Institute of Freshwater Fisheries, Huzhou, China; ^2^ Development Center of Huzhou Agricultural Science and Technology, Huzhou, China; ^3^ College of Animal Science and Technology, Jilin Agricultural University, Changchun, China

**Keywords:** largemouth bass iridovirus, *Pichia pastoris*, immune protection, oral vaccines, presentation-related genes, immunoglobulins

## Abstract

Largemouth bass iridovirus (LMBV) can cause high mortality and lead to heavy economic loss in the cultivation of largemouth bass, but there was no effective treatment. Here, the present study constructed a recombinant *Pichia pastoris* expressing LMBV major capsid protein (*MCPD*). The recombinant GS115-pW317-*MCPD* was then used to immunize largemouth bass *via* oral administration, and mucosal immune response mediated by immunoglobulins (Igs) was measured after oral immunization. Serum antibody levels were measured by ELISA, neutralizing antibody titers were determined by serum neutralization test (SNT), antigen presentation-related gene expressions were detected by RT-PCR, and the histopathological characteristics of immunized fish were assessed after challenging with 0.1 ml 10^7.19^ TCID_50_/ml LMBV. The relative percentage survival (RPS) was also determined. Our results showed that the serum antibody titers of immunized fish were significantly higher than that of control groups (P < 0.05). IgT and IgM expressions in gut were increased significantly after vaccination with GS115-pW317-*MCPD*; however, much stronger response in gut was observed as compared with gill. The expression levels of major histocompatibility complex (*MHC*) II, *CD*8, and T-cell receptor (*TCR*) were significantly elevated in GS115-pW317-*MCPD* group (P < 0.05), while *CD*4 and *MHC* I transcription levels remained unchanged after oral immunization (*P* > 0.05). The RPS of fish orally immunized with 1.0 × 10^8^ CFU/g GS115-pW317-*MCPD* was reached up to 41.6% after challenge with 0.1 ml 10^9.46^ TCID_50_/ml LMBV. Moreover, orally immunizing with GS115-pW317-*MCPD* can relieve the pathological damage caused by LMBV. Therefore, GS115-pW317-*MCPD* showed a promising potential against LMBV.

## 1 Introduction

Largemouth bass (*Micropterus salmoides*) was a widely cultured freshwater fish in China (1, 2). Since 2009, it has been suffering an outbreak of iridovirus disease, which was caused by largemouth bass virus (LMBV) (3). Frequent outbreaks of the disease may hinder the development of largemouth bass aquatic industry (4), so there are urgent needs for effective ways to control the disease.

Currently, there are still no effective chemical drugs used to control the outbreak of LMBV; also, drug residues, food, and environmental safety caused by excessive use of drugs are other disadvantages of chemotherapy (5). Vaccine is the most effective tool to prevent and control fish diseases; it was also considered as an effective way to reduce the use of chemical drugs (6). According to the preparation method of the vaccine, it can be divided into inactivated vaccine, live vaccine, and subunit and biotechnology vaccine. There are three main vaccine delivery ways in fish: injection, immersion, and oral administration, among which injection was the most effective way. However, injection was not suitable for large-scale operation in aquaculture; meanwhile, it could hamper the growth of immunized aquatic animals (7, 8). Immersion was another common immunization route, but a low immune effect limited its use in aquaculture. So, the urgent need of new methods for fish immunization has become the consensus of the aquaculture industry and academia. More and more effort has been made to pursue more convenient and efficacious immune methods to control fish disease (9, 10). Oral immunization has become the focus research topic due to its convenience in actual production (11). The greatest advantage of oral immunization was that it can trigger mucosal and systemic immune responses *via* activated dendritic cells (DCs) (12, 13), which then metastasizes to mesenteric lymph nodes (MLNs) and presents processed antigens to T and B lymphocytes. On the other hand, it is easy to elicit a rapid local innate immune response in the intestine.

Yeast is an ideal protein preparation platform; it was easy to culture and suitable for large-scale production. Additionally, β-glucan, the main compound of the cell wall of yeast, was recognized as an immune-stimulant supplement in fish, which made the yeast an attractive candidate for antigen delivery to the intestinal mucosa (14). On the other hand, it is very easy to administer with no stress to fish of various sizes and ages. In this study, *Pichia pastoris* GS115 was used as a host to express the LMBV major capsid protein (MCP) and introduced into *P. pastoris* GS115 by electroporation. Then, orally immunized to largemouth bass. Serum antibody levels of fish immunized with recombinant GS115-pW317-*MCPD* were measured by ELISA, neutralizing antibody titers were determined by SNT, and immune response of immunoglobulins (Igs) and antigen presentation-related gene expressions were detected by RT-PCR. The relative percentage survival (RPS) and the histopathological characteristics of immunized fish were also assessed.

## 2 Materials and Methods

### 2.1 Fish

Healthy largemouth bass (24.6 ± 3.1 g) were purchased from Zhejiang Institute of Freshwater Fisheries Comprehensive Experimental Base (Zhejiang, China) and were acclimatized in recirculating aquaculture system for 14 days. Before the experiment, fish were randomly sampled for the examination of bacteria, parasite (microexamination), and virus (PCR for LMBV); no pathogens were detected, and no naturally dead fish were found during the temporary cultivation. Water quality conditions were controlled by recirculating the aquaculture system as follows: temperature: 28°C ± 1°C, dissolved oxygen >5 mg l^-1^; nitrites < 0.02 mg l^-1^; ammonia < 0.1 mg l^-1^.

### 2.2 Virus and Cell Lines

The LMBV was originally isolated and identified from diseased largemouth bass by our laboratory. Fathead minnow (FHM) cell was obtained from Pearl River Fisheries Research Institute and grown at 28°C in Dulbecco’s Modified Eagle Medium (DMEM) supplemented with 10% fetal bovine serum. Epithelioma papulosum cyprini (EPC) cells were maintained in our lab and grown at 28°C in DMEM. The *P. pastoris* GS115 was obtained from Hangzhou Fenghai Biotechnology Co., Ltd.

### 2.3 Codon Optimization and Gene Synthesis

Major epitope regions of *MCPD* protein were analyzed and optimized (see in the attachment). In order to facilitate the purification of protein and antibody level determination, hexa-histidine tag was designed. The *MCPD* gene (hexa-histidine tag at the N-terminus) was synthesized by Sangon Biotech (Shanghai, China) Co., Ltd.

### 2.4 Construction of Recombinant GS115-pW317-*MCPD*

**Figure 1:**
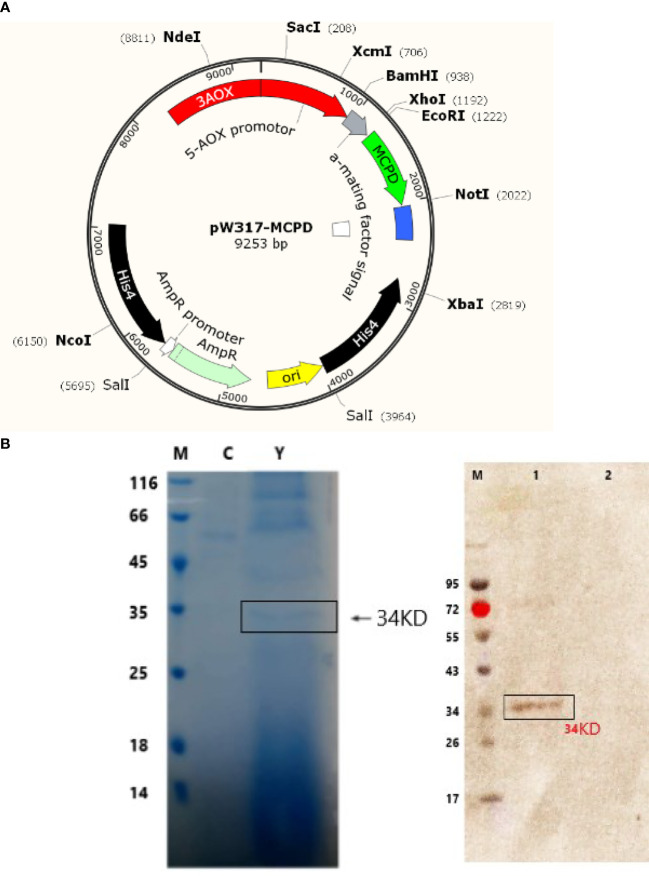
Sodium dodecyl sulfate polyacrylamide gel electrophoresis (SDS- PAGE) and Western blot detection of recombinant truncated *MCPD* protein expression. **(A)** Plasmid map of pW317-*MCPD*. **(B)** SDS-PAGE and Western blot detection. M, protein standard molecular weight; C, blank control; Y, recombinant bacteria; 1, recombinant bacteria; 2, blank control.

The primers for amplifying *MCPD* were listed as follows: F : AATTGGTTTGACTAATTCCATAAT; R : AATGTTCGTCAAAATGGTGAC. EcoR I and NotI sites were also added, respectively (**Figure 1**). The pWB17 plasmid and the recombinant pWB17-*MCPD* were integrated into *P. pastoris* GS115 by electroporation. Recombinant GS115-pW317-*MCPD* and GS115-pW317 were cultured in yeast extract peptone dextrose (YPD, containing 1,000 µg/ml zeocin) medium at 37°C.

### 2.5 Identification of Recombinant GS115-pW317-*MCPD*

After induction for 24 h, recombinant GS115-pW317-*MCPD* cells were harvested and then disrupted by sonication. The protein concentration of the culture lysates was then determined. The *MCPD* proteins were separated by Sodium Dodecyl Sulfate Polyacrylamide Gel Electrophoresis and then recombinant *MCPD* proteins (with His-Tag) were identified by Western blotting using anti-His-tag monoclonal antibody and Horseradish Peroxidase (RRP)-conjugated goat anti-mouse IgG as antibodies (Beijing Solarbio Science & Technology Co., Ltd.).

### 2.6 Assessment of Immune Efficacy of Recombinant GS115-pW317-*MCPD*

#### 2.6.1 Oral Immunization and Experimental Design

Five groups of fish (30 each) were orally immunized with 10^8^ CFU/g GS115-pW317-*MCPD* (commercial basal diet + GS115-pW317-*MCPD*), 10^7^ CFU/g GS115-pW317-*MCPD* (commercial basal diet + GS115-pW317-*MCPD*), GS115-pW317 (10^8^ CFU/g and 10^7^ CFU/g), and PBS (commercial basal diet+PBS). Briefly, the harvested GS115-pW317-*MCPD* and GS115-pW317 were washed twice with PBS, and then the concentrations of harvested GS115-pW317-*MCPD* and GS115-pW317 were determined, and the required amount of GS115-pW317-*MCPD* and GS115-pW317 was mixed with commercial basal diet for oral immunization. All fish were fed twice for 1 day at 08:00 and 16:00 h, respectively. The feeding trial was conducted for 2 weeks. Sera were collected from the caudal vein of each fish for antibody titer determination. Gill, gut, and kidney were sampled on days 7, 14, 21, 28, and 35 for immune response and gene expression assays (3 fish/time point). All treatments and control groups were conducted with three replicates (30 fish for each).

#### 2.6.2 Detection of Anti-*MCPD* Antibody

The antibody levels of immunized fish were determined by ELISA according to previous studies (6, 10). Three largemouth bass were randomly selected from each group, serum from each fish was obtained and then diluted by 50 mM Na_2_CO_3_/NaHCO_3_ buffer (pH 9.6) at 37°C for 4 h (each conducted with three parallel repetitions). Purified *MCPD* protein (with His-Tag) was then added into 96-microtiter plate at 37°C; after incubation for 1 h, each well was washed with Phosphate Buffered Saline with 0.05% Tween (PBST) 3 times. Here, 100 µl of McAb Mouse Anti-His Tag (1:2,500) used as primary antibody were then added into each well of plate at 37°C for 1 h, then washed with PBST buffer 3 times. Subsequently, HRP-conjugated goat-mouse IgG antibody (1:2,000) used as secondary antibody was added into each well of plate at 37°C; after incubation for 1 h, each well was washed by PBST buffer 3 times, then 2 mol/L H_2_SO_4_ were used to stop the reaction; Tetramethylbenzidine (TMB) was used to color the result.

#### 2.6.3 Immune Response of Immunoglobulins

Gene expression levels of IgM and IgT after immunization were conducted. Total RNA extracted from each tissue (gill, gut, and kidney) was prepared by TRIzol lysis (Invitrogen, USA). cDNA was then synthesized according to the protocol of Reverse Transcriptase M-MLV Kit (TaKaRa, Dalian, China). The RT-PCR was performed using THUNDERBIRD SYBR qPCR Mix Kit (TOYOBO, Shanghai, China). The primer was designed following previous reports (15, 16).

#### 2.6.4 Antigen Presentation-Related Gene Expression

Expression levels of antigen presentation-related genes major histocompatibility complex (*MHC*) I, *MHC* II, *CD*8, *CD*4, and T-cell receptor (*TCR*) were also determined by RT-PCR. Briefly, total RNA was extracted from gill, gut, and kidney by TRIzol Reagent (Cwbio, Beijing, China). The RT-PCR was performed using THUNDERBIRD SYBR qPCR Mix Kit (TOYOBO, Shanghai, China) and carried out in a Stratagene MxPro System (Stratagene mx3005p, USA) in 96-well reaction plates.

#### 2.6.5 Serum Neutralization Test

Neutralizing antibody titers were determined by serum neutralization test (SNT) in EPC cells according to previous methods (17). Firstly, harvested sera were doubly serially diluted in DMEM (without Fatal Bovine Serun), and then 50 μl of serum was mixed with the same volume of DMEM and 100 tissue TCID_50_ of virus. Secondly, 100 μl preincubated mixture (25°C for 1 h) were added to EPC cell monolayers. Finally, 5 days after incubation, the highest dilution at which 50% EPC cells were inhibited was considered as the LMBV neutralizing antibody titer.

#### 2.6.6 Histological Evaluation

At 35 days post immunization (dpi), three largemouth bass were randomly selected from group GS115-pW317-*MCPD* and GS115-pW317 and injected 0.1 ml 10^8.66^ TCID_50_/30 ml LMBV per fish, six largemouth bass were randomly selected from PBS group, three fish were challenged with 0.1 ml 10^7.19^ TCID_50_/ml LMBV, the remaining three fish were treated with no virus. After 14 days, the liver, kidney, and spleen samples in each group were received, fixed in 10% formalin, embedded in paraffin wax, and sectioned by microtome, and then hematoxylin and eosin (H&E) staining was conducted to analyze the histological change of immunized fish.

### 2.7 Challenge Test

Five groups were set in challenge test. Each group contains 20 largemouth bass; each group was performed with three replicates. Immunized groups were orally immunized with GS115-pW317-*MCPD* (commercial basal diet + GS115-pW317-*MCPD* 10^7^ CFU/g feed) and GS115-pW317-*MCPD* (commercial basal diet + GS115-pW317-*MCPD* 10^8^ CFU/g feed). GS115-pW317 (10^7^ CFU/g feed and 10^8^ CFU/g) and PBS (commercial basal diet+PBS) served as control group. All fish were fed twice a day and lasted for 2 weeks. On 21 dpi, all fish were challenged with 0.1 ml 10^9.46^ TCID_50_/ml LMBV per fish. Mortalities in each group were recorded daily for 15 days. The RPS was calculated as follows:


RPS (%)=(mortality of immunized group%−mortality of control group%)/mortality of control group%.

### 2.8 Data Analysis

The data were expressed as the mean and standard deviation (mean ± SD) and analyzed by Statistical Product and Service Solutions (SPSS 19.0). The difference was considered statistically significant when P < 0.05 and highly significant when P < 0.01.

## 3 Results

### 3.1 Construction of Recombinant GS115-pW317-*MCPD*

GS115-pW317-*MCPD* (**Figure 1A**) was constructed and introduced into *P. pastoris* GS115 by electroporation. The cell lysates GS115-pW317-*MCPD* and GS115-pW317 were subjected to SDS-PAGE and Western blot. A specific band of about 43 kDa was detected in GS115-pW317-*MCPD* (**Figure 1B**), but no band was found in GS115-pW317 (**Figure 1B**), which indicated that *MCPD* was successfully expressed in *P. pastoris* GS115.

### 3.2 Assessment of Immune Efficacy of Recombinant GS115-pW317-*MCPD*

#### 3.2.1 Detection of Anti-*MCPD* Antibody

**Figure 2:**
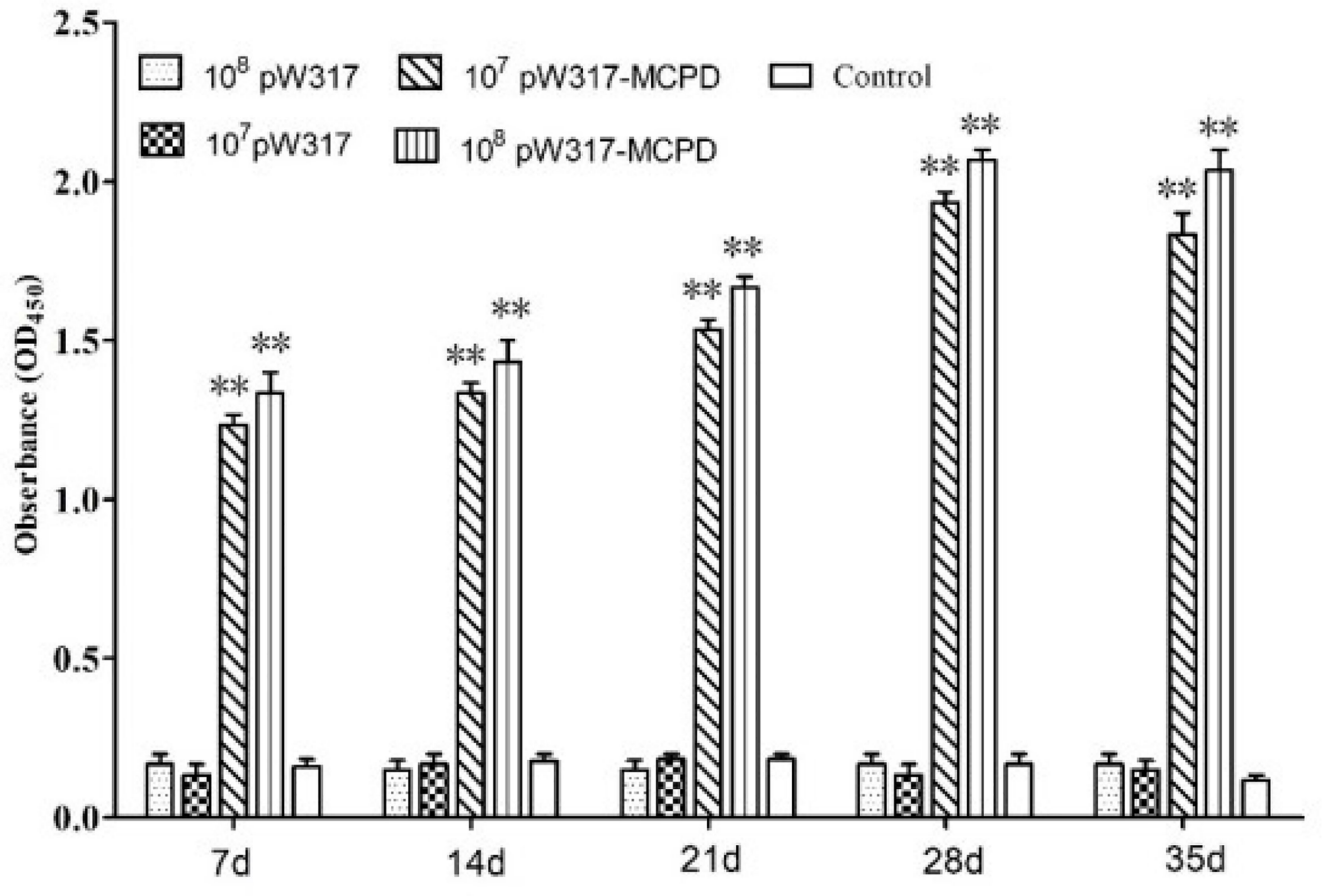
The levels of serum antibody. Serum was collected from the fish at 7, 14, 21, 28, and 35 dpi, and serum antibodies against MCPD were determined by ELISA. Three replicates were set for the tests, with three fish per replicate. Data are means for three assays and presented as the means ± SE. **P < 0.01. 10^7^pW317: 10^7^ CFU/g GS115-pW317; 10^8^pW317: 10^8^ CFU/g GS115-pW317; 10^7^pW317-MCPD: 10^7^ CFU/g GS115-pW317-MCPD; 10^8^ pW317-MCPD: 10^8^ CFU/g GS115-pW317-MCPD.

Results from ELISA showed that the antibody levels in GS115-pW317-*MCPD*-immunized group were significantly higher than those of fish fed GS115-pW317 or PBS (*P* < 0.01). Serum antibody levels in 10^8^ CFU/g GS115-pW317-*MCPD*-immunized group were about 8-fold higher than those in the PBS group and GS115-pW317 group (**Figure 2**); the highest antibody level was detected on 28 dpi.

#### 3.2.2 Immune Response of Immunoglobulins

The expression levels of IgM in all tested tissues were significantly increased after oral immunization. After oral immunization, IgM had highest expression in the kidney followed by gut. The expression level of IgM in the kidney increased firstly and then decreased slowly, reached a peak at 28 dpi, which was about 11-fold higher than that in the PBS group. Interestingly, the IgM mRNA level in GS115-pW317 group was significantly higher than that in the PBS group in gut and head kidney at 21, 28, and 35 dpi (P < 0.05).

**Figure 3:**
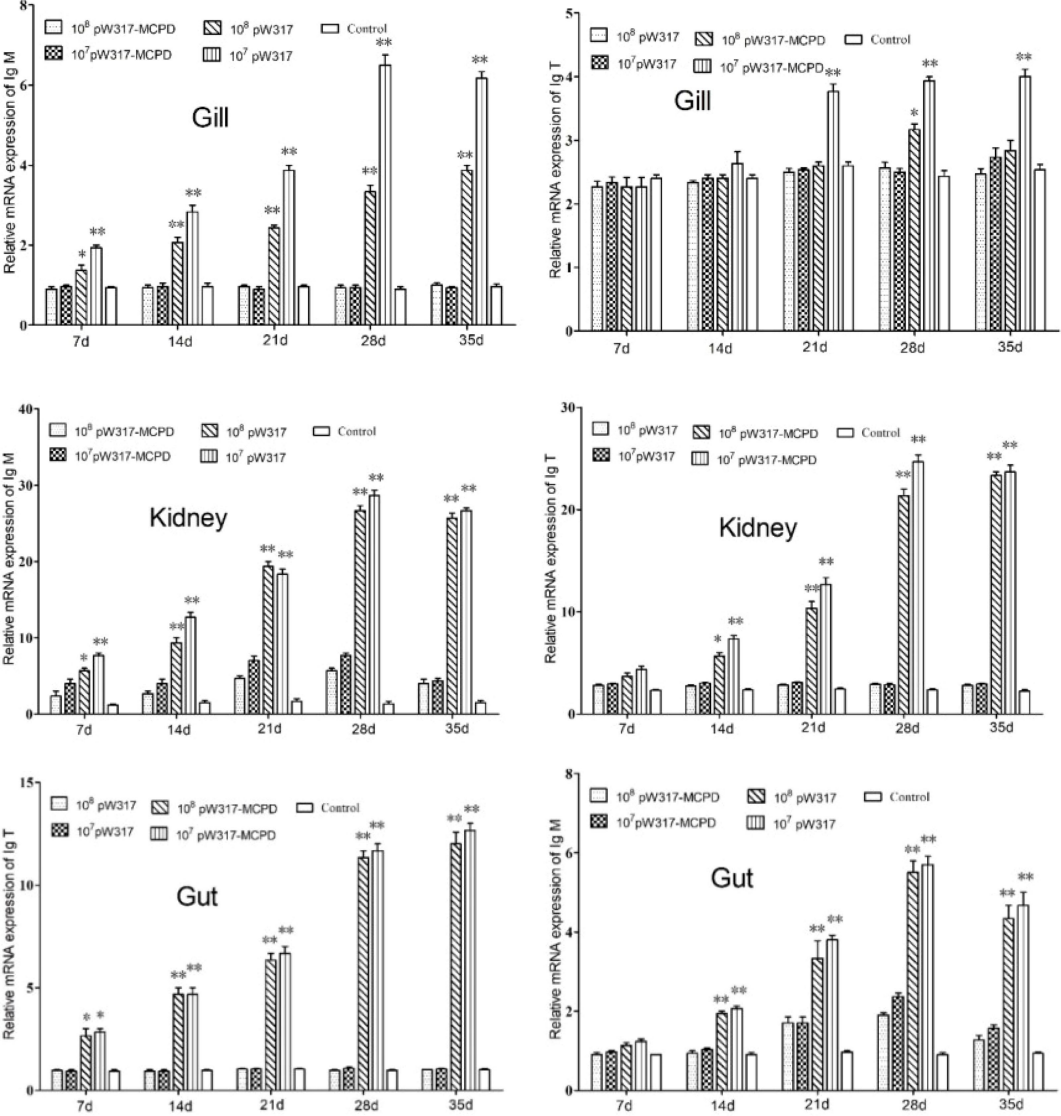
The expression level of IgM and IgT in different tissues after immunization. 10^7^pW317: 10^7^ CFU/g GS115-pW317; 10^8^pW317: 10^8^ CFU/g GS115-pW317; 10^7^pW317-MCPD: 10^7^ CFU/g GS115-pW317-MCPD; 10^8^ pW317-MCPD: 10^8^ CFU/g GS115-pW317-MCPD. **P < 0.01; *P < 0.05.

Compared with GS115-pW317 and PBS control groups, IgT was highly expressed in head kidney and gut. In head kidney, the IgT gene expression reached its peak at 21 dpi, which was about 12-fold higher than that in the control PBS group; the corresponding data in gut was 5.5-fold. However, much stronger response in gut was observed as compared with gill after oral immunization (**Figure 3**).

#### 3.2.3 Antigen Presentation-Related Gene Expression

**Figure 4:**
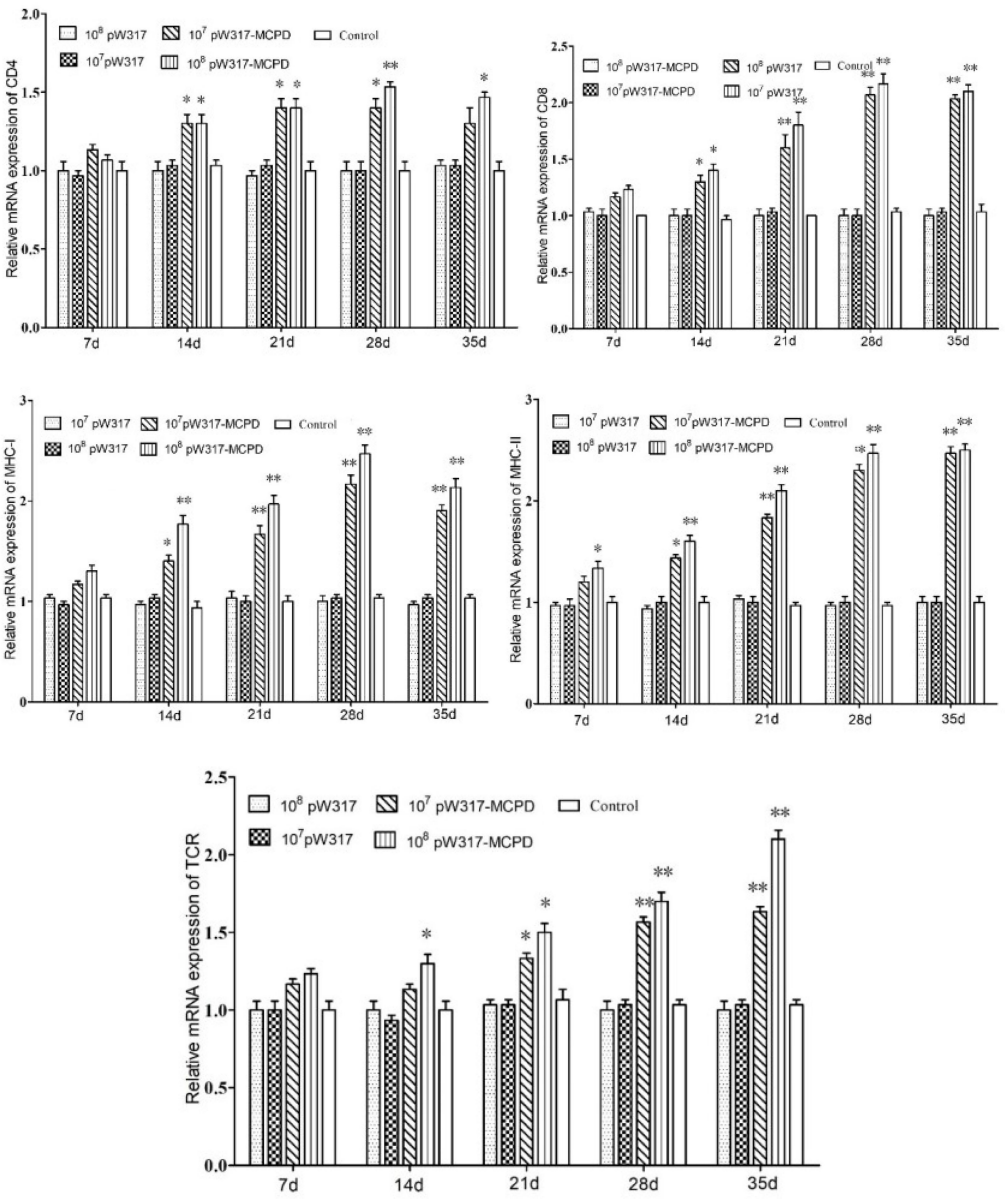
The expression level of antigen presentation-related gene in gill after immunization. 10^7^pW317: 10^7^ CFU/g GS115-pW317; 10^8^pW317: 10^8^ CFU/g GS115-pW317; 10^7^pW317-MCPD: 10^7^ CFU/g GS115-pW317-MCPD; 10^8^ pW317-MCPD: 10^8^ CFU/g GS115-pW317-MCPD. **P < 0.01; *P < 0.05.

**Figure 5:**
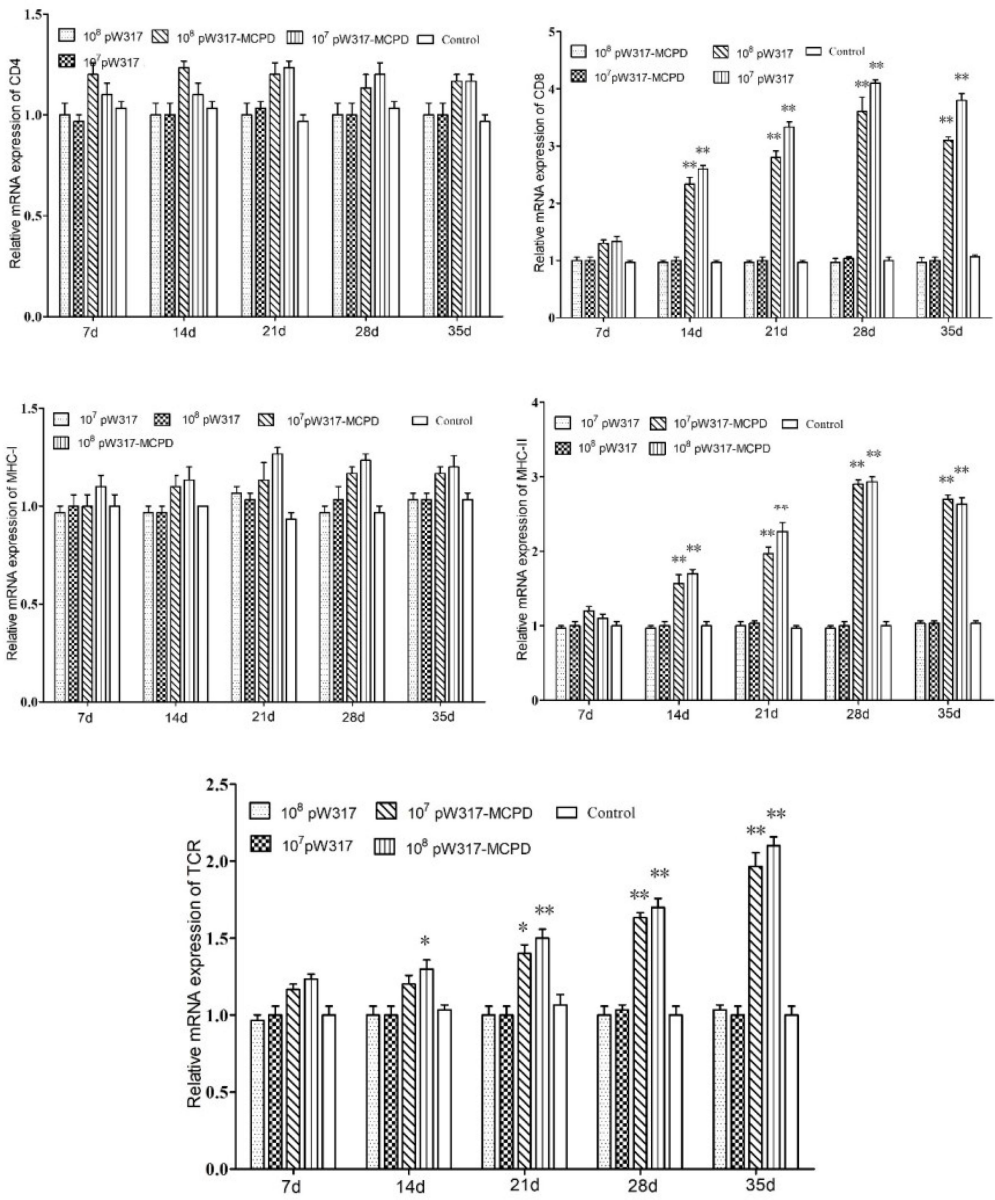
The expression level of antigen presentation-related gene in gut after immunization. 10^7^pW317: 10^7^ CFU/g GS115-pW317; 10^8^pW317: 10^8^ CFU/g GS115-pW317; 10^7^pW317-MCPD: 10^7^ CFU/g GS115-pW317-MCPD; 10^8^ pW317-MCPD: 10^8^ CFU/g GS115-pW317-MCPD. **P < 0.01; *P < 0.05.

**Figure 6:**
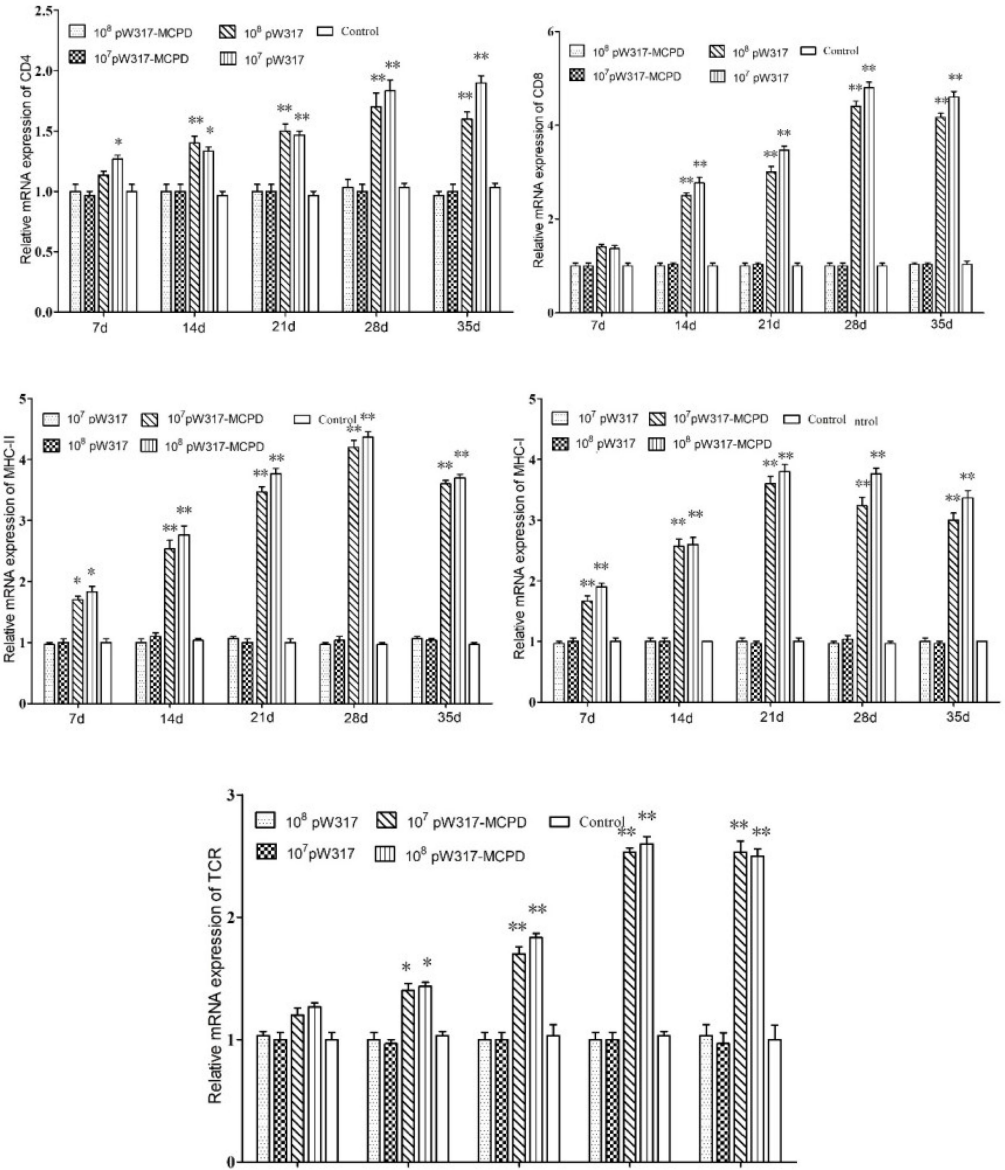
The expression level of antigen presentation-related gene in head kidney after immunization. 10^7^pW317: 10^7^ CFU/g GS115-pW317; 10^8^pW317: 10^8^ CFU/g GS115-pW317; 10^7^pW317-MCPD: 10^7^ CFU/g GS115-pW317-MCPD; 10^8^ pW317-MCPD: 10^8^ CFU/g GS115-pW317-MCPD. **P < 0.01; *P < 0.05.

The RT-PCR analysis showed that the expressions of *MHC*-I mRNA in the tested tissues were increased as compared with those in the control group after oral immunization. In gill, the expressions of *MHC*-I mRNA in GS115-pW317-*MCPD*-immunized group were significantly upregulated and reached the top at 21 dpi, which was 2.4-fold higher than those in the PBS groups (**Figure 4**). However, no difference was found in gut tissue (**Figure 5**). The peak value of kidney tissue was 3.8 times higher than that of the PBS group on 28 dpi (**Figure 6**).

The mRNA expressions of *MHC* II in GS115-pW317-*MCPD*-immunized group were significantly upregulated as compared with those in the control group (P < 0.01). In kidney, the expression level of *MHC* II reached up to a peak at 21 dpi, which was 2.4-fold higher than that in the PBS group. In gut, the peak value of 10^8^ CFU/g group was found at 21 dpi, which was 4.5-fold higher than that in the PBS group (**Figure 5**).

The results of mRNA expressions of *CD*4 in kidney, gut, and gill were shown in **Figures 4** **–** **6**. In kidney, the relative expression of *CD*4 transcript level in the immunized group was significantly upregulated and reached its peak at dpi 28; similar results were also detected in gill. However, in gut, both immunized groups showed no difference compared with PBS group and empty carrier group (P > 0.05).

The mRNA expressions of *CD*8 in kidney, gut, and gill were significantly increased as compared with those in the control group (P < 0.01) (**Figure 4**). In kidney, the expressions of *CD*8 mRNA were significantly upregulated at 28 dpi as compared with those in the PBS group (P < 0.01) (**Figure 6**). In gut, the peak value of 10^8^ CFU/g group was found at 28 dpi, which was 4.2-fold higher than that in the PBS group (**Figure 5**).

The RT-PCR analysis showed that the expressions of *TCR* mRNA in the three tested tissues were increased as compared with those in the control group. The peak value of kidney tissue was 2.6 times higher than that of the PBS group on 28 dpi (P < 0.01). In gill, the expressions of *TCR* mRNA reached up to a peak at 35 dpi, with a peak value of 2.2-fold higher than that of the PBS group (**Figure 4**).

#### 3.2.4 Histopathological Analysis

**Figure 7:**
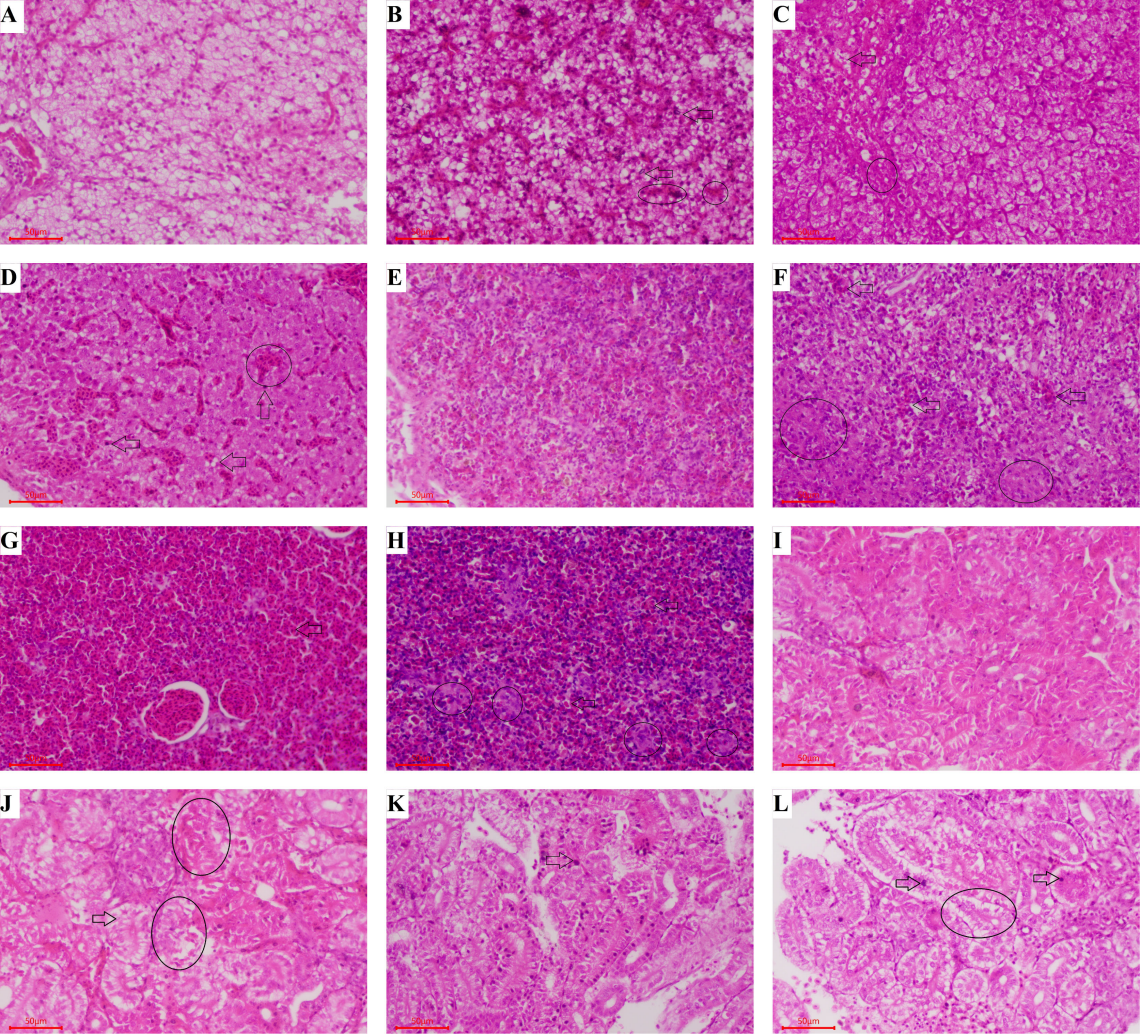
Histopathological assessment of liver, spleen, and kidney from largemouth bass after challenge. Groups A, E, and I: Fed with commercial basal diet+PBS, treated with no virus; Groups B, F, and J: Fed with commercial basal diet+10^7^ CFU/g GS115-pW317-MCPD, challenged with LMBV; Groups C, G, and K: Fed with commercial basal diet+10^8^ CFU/g GS115-pW317-MCPD, challenged with LMBV; Groups D, H, and L: Fed with commercial basal diet+PBS, challenged with LMBV. **(A)** The structure of liver cell was integrated, and the gland was clearly visible. **(B)** Some hepatocytes were swollen and degenerated (⇨), the chromatin edges are concentrated and moved into rings (O). **(C)** Hepatic cell swelling and vesicular degeneration were detected (⇨), the chromatin edges are concentrated and moved into rings (O). **(D)** Hepatic sinus dilatation and congestion (⇨), hepatic cell swelling and vacuolar degeneration and steatosis were found (O). **(E)** The spleen cells were normal with intact structure, and no pathological changes were found. **(F)** The splenic sinus was diluted and filled with pink serous fluid, a small amount of reticular cellulose and a large number of red blood cells were detected (⇨), spleen structure is disordered and reticular cells increase (O). **(G)** Some congestive spleen cells were found (⇨). **(H)** Spleen sinuses were dilated and congested (O), a large number of red blood cells were detected in the spleen tissue (⇨). **(I)** The kidney cells were normal with intact structure. **(J)** Swelling and degeneration of renal tubular epithelial cells were detected (⇨), tubule epithelial cells were swollen and separated from the basal side of the renal tubules (O). **(K)** Some swelling and degeneration of renal tubular epithelial cells were detected (⇨). **(L)** Tubule epithelial cells were highly swollen (⇨) and partially separated from the basal side of the renal tubules (O).

Varying degrees of cytopathic lesions were detected in largemouth bass after challenging with LMBV, however, in the control group treated with non virus, cells were normal with intact structure, and no pathological changes were found (**Figures 7A, E, I**). Hepatic sinus dilatation and congestion, hepatic cell swelling and vacuolar degeneration, and steatosis were found in the liver of largemouth bass after challenging with LMBV (**Figures 7B–D**). The splenic sinus was diluted and filled with pink serous fluid, and a large number of red blood cells were detected in the spleen (**Figures 7F–H**). Tubule epithelial cells were highly swollen and partially separated from the basal side of the renal tubules (**Figures 7 J–L** **)**. However, the pathological injuries in spleen and kidney were relatively light when immunized with 10^8^ CFU/g GS115-pW317-*MCPD*.

#### 3.2.5 Serum Neutralization Test

**Figure 8:**
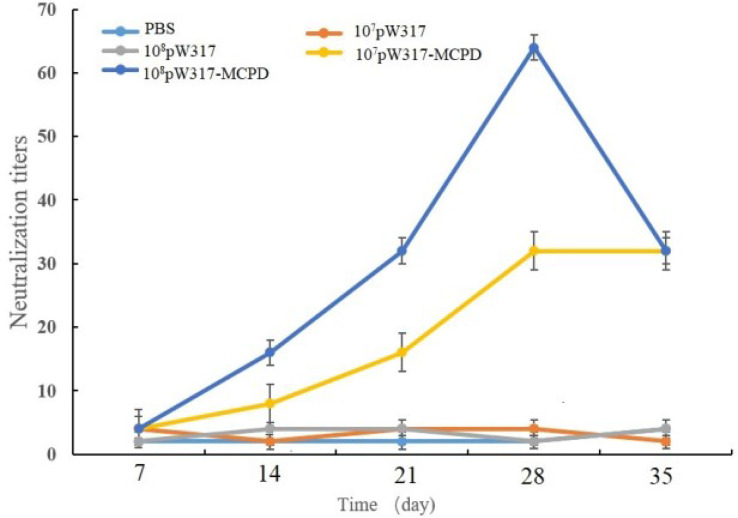
Serum neutralization assay of largemouth bass after oral immunization. Note: 10^7^pW317: 10^7^ CFU/g GS115-pW317; 10^8^pW317: 10^8^ CFU/g GS115-pW317; 10^7^pW317-MCPD: 10^7^ CFU/g GS115-pW317-MCPD; 10^8^ pW317-MCPD: 10^8^ CFU/g GS115-pW317-MCPD.

The results of SNT were shown in **Figure 8**. Antibody titers in 10^8^ CFU/g GS115-pW317-*MCPD*-immunized group were significantly higher than those in GS115-pW317 and PBS groups; the peak titer in 10^8^ CFU/g GS115-pW317-*MCPD* was 1:64 at dpi 28, while the peak titer in 10^7^ CFU/g group was 1:32.

### 3.3 Challenge Test

**Figure 9:**
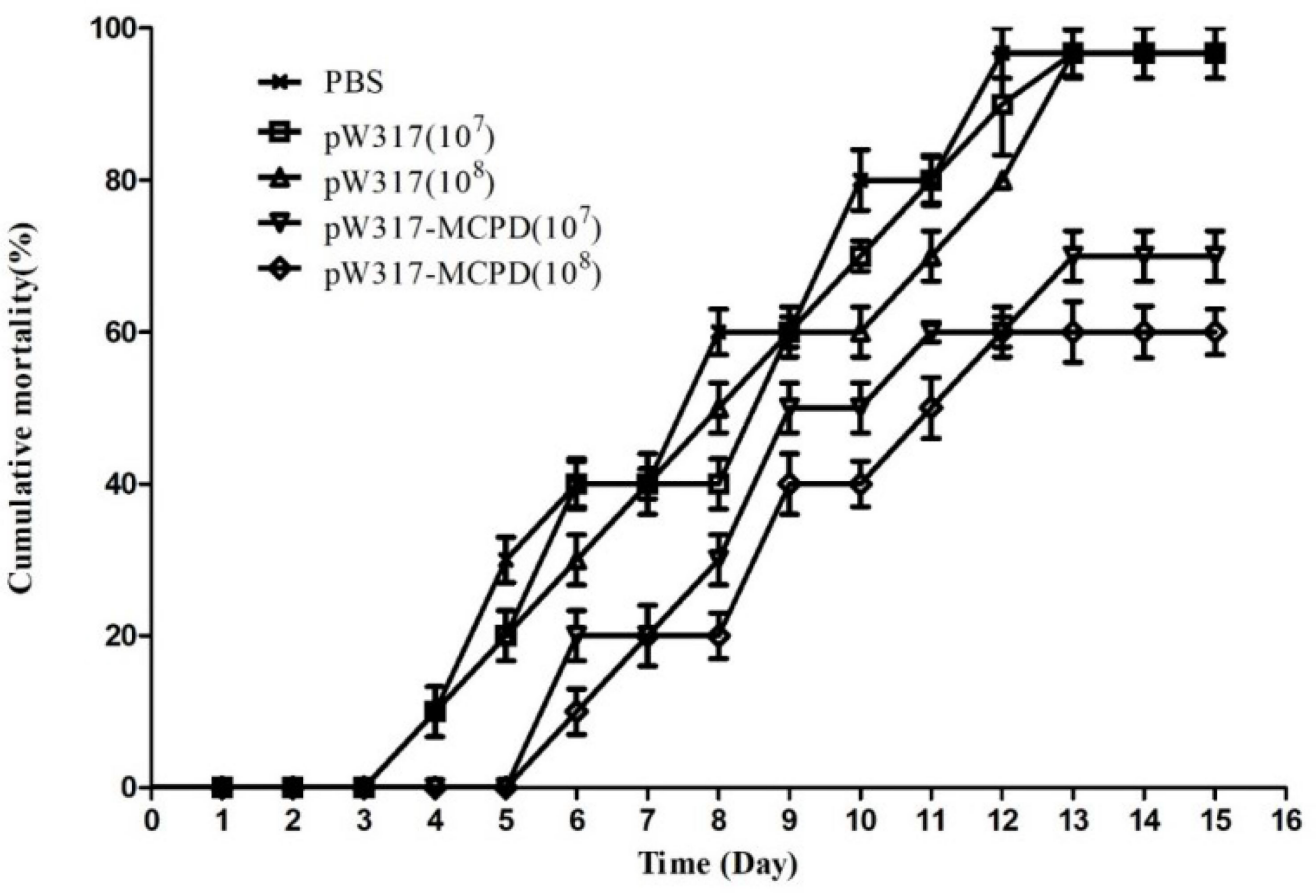
The cumulative mortality of vaccinated largemouth bass. Note: 10^7^pW317: 10^7^ CFU/g GS115-pW317; 10^8^pW317: 10^8^ CFU/g GS115-pW317; 10^7^pW317-MCPD: 10^7^ CFU/g GS115-pW317-MCPD; 10^8^ pW317-MCPD: 10^8^ CFU/g GS115-pW317-MCPD.

The challenged largemouth bass began to die on day 4 in PBS group; 96.7% mortality was found in GS115-pW317 and PBS groups. However, the immunized group began to die on day 6; the mortality of fish immunized with both doses of pW317-*MCPD* was significantly reduced (P < 0.01) (**Figure 9**). The RPS of 10^8^ CFU/g GS115-pW317-*MCPD* and 10^7^ CFU/g GS115-pW317-*MCPD* was 41.6% and 33.3%, respectively.

## 4 Discussion

LMBV was one of the most harmful viruses in the cultivation of largemouth bass. However, there is still no effective drug to treat LMBV infection. Vaccination is considered as an effective method to control the outbreak of fish disease (18). The route of immunization plays a vital role in the effectiveness of vaccine. There were three main immunization routes in aquaculture: bath/immersion, injection, and oral administration (19). Oral immunization was the preference because it can be used for mass vaccination, and it is relatively easy and stress-free. In the present study, an oral vaccine that expressed the *MCP* protein of LMBV was constructed *via* the yeast *P. pastoris*, and the results from the challenge test showed that GS115-pW317-*MCPD* can effectively elicit immunity response and protect largemouth bass against LMBV, which indicated that *P. pastoris* could become a promising oral vaccine carrier against fish virus.

Neutralizing antibody plays a vital role in defending against viral diseases of fish (12, 20). Results from the present study showed that the antibody titers reached up to 64 at 28 dpi, and the RPS of fish orally immunized with GS115-pW317-*MCPD* was 41.6%. However, Yi et al. (21) reported that SN antibody titers could reach a peak with the value of 1:375 ± 40 at 14 days after immunization *via* injection, which was almost 6-fold higher than our results. This may be because the immunological effect of injection was better than oral administration. Moreover, the content of antigen expressed by *P. pastoris* may not be enough for largemouth bass. So, more technology applied to improve immune efficacy is needed. Coexpression of antigen or some adjuvant-related protein can enhance the immune effect. Liu et al. (22) found that coexpression of influenza NP-M2 and a mucosal adjuvant (FliC) by *Lactobacillus plantarum* could enhance the protective immune responses against H9N2 influenza. Recent research demonstrated that coexpression of some invasion-related proteins can improve the immune effect. Fibronectin-binding proteins (FnBPA) were one of the most studied invasion-related proteins; it could invade mammalian cells by binding to the α5β1 integrin on the surface of the host cell membrane. Therefore, it was usually used to carry DNA to host cells (23). Liu et al. (24) confirmed that invasive *L. plantarum* expressing the FnBPA protein enhanced humoral and cellular immunity and improved protective effectiveness against *Eimeria tenella*. Xue et al. (25) used the invasive *L. plantarum* (expressing FnBPA) as a live bacterial vector to coexpress *Trichinella spiralis* SS1 and murine interleukin-4; *in vivo* results exhibited that FnBPA increased the effect of antigen presentation and immune protection. Additional studies are required to further coexpress more antigens; some adjuvant or invasion-related proteins are needed to enhance the humoral and cellular immunity (26).

Igs are key element components of the immune response in teleost fish, playing a crucial role in protecting fish against various pathogens. So far, three major classes of Igs, IgM, IgD, and IgT/Z, were found in teleost fish (27). IgT, equivalent to that of mammalian IgA, was firstly detected in rainbow trout (*Oncorhynchus mykiss*) (28, 29). More and more research showed that IgT plays a key role in the mucosal immunity of teleost (28, 29). IgM is the best characterized teleost Ig isotype and plays an important role in systemic immunity (30). The expression levels of IgM in kidney, gut, and gill were significantly increased after oral immunization of GS115-pW317-*MCPD* and highest expressed in kidney followed by gut. Interestingly, the IgM mRNA level in GS115-pW317 group was significantly higher than that in the PBS group in gut and head kidney. This may be because the main cell wall component of yeast, β-glucan, has been characterized as a dietary immune-stimulant supplement that could increase complement activity and enhance the expression of IgM (14). As for IgT, after administering recombinant GS115-pW317-*MCPD*, it was highly expressed in the head kidney and gut largemouth bass. Similar to our results, the expression level of IgT in largemouth bass was highest expressed in head kidney after immersion vaccination with *Aeromonas hydrophila*, then in spleen, liver, and gill. By contrast, the highest expression level of Ig tau heavy chain (IgT) in turbot and flounder was detected in gill and spleen (31, 32). Because IgT may exhibit fish species specificity (30). Our results also indicated that much stronger response of IgT was observed in gut after oral immunization; higher levels of IgT expression were observed in gut than in gill, which indicated that gut may be the main mucosal immunity site. Some studies have shown that sIgT and IgT+ cells in trout develop a stronger mucosal immune response than systemic immunity that indicated that IgT presented played a major role in mucosal immunity (28, 29). Based on the above, we speculated that intestinal tract plays an important role in mucosal immunity after oral immunization; also, IgT played a major important role in largemouth bass mucosal immunity.

Antigen handling and presentation play an important role in adaptive immunity. When the antigen binds to *MHC* I and II, antigens are processed and presented to specific lymphocytes (33). *MHC* is a group of cell surface proteins that interact with T cells in the acquired immune system through *TCR* (34) and is a key protein for recognizing invading pathogens and stimulating immune responses. *MHC* I and *MHC* II recognize antigenic peptides mainly *via TCR* especially the protein chains on *TCR*-A and *TCR*-B (35). *MHC* I is an exogenous peptide produced by degradation of intracellular pathogens into cytotoxic *CD*8^+^ T cells; additionally, *MHC* I can recognize exogenous antigens and present to cytotoxic T cells through a mechanism called antigenic cross presentation (36). Some reports showed that *MHC* II plays an important role in presenting the extracellular antigen to *CD*4^+^ T cells (37). The results from the present study showed that *MHC* II b, *CD*8, and *TCR* were elevated, while *CD*4 and *MHC* I transcription levels remained unchanged after oral immunized with GS115-pW317-*MCPD*. Consistent with our research, Pichietti et al. (38) reported that the expression of *CD*8-a in posterior intestine was significantly elevated after immunization, while the expression of *TCR*-b and *CD*8-a was higher than that of *CD*4. By contrast, after immunizing with inactivated viruses *via* immersion and oral administration, the expression levels of *MHC* I and *CD*8 mRNA were significantly upregulated in *Dicentrarchus labrax* and *Epinephelus coioides* (39–41); this indicated that a subset of *CD*8-aþ T cells could bind antigens out of the context of *MHC* molecules that was also found in mammalian *TCR*-gd *CD*8-aaþ IEL subset, which means that *MHC*-restricted peptide presentation was not absolutely required (42).

In conclusion, an oral vaccine using *P. pastoris* that expressed *MCPD* has been developed to control LMBV. Our findings revealed that the *P. pastoris* yeast expression system was a promising vehicle for antigen delivery; it showed a good prospect in the application of fish oral vaccine. However, the mucosal immune mechanism should be included in future studies.

## Data Availability Statement

The original contributions presented in the study are included in the article/**Supplementary Material**. Further inquiries can be directed to the corresponding authors.

## Ethics Statement

The animal study was reviewed and approved according to the guidelines of the Animal Experiment Committee, Zhejiang Institute of Freshwater Fishery (ZJIFF20210302).

## Author Contributions

JY, GY, WZ and HZ contributed to the conception of the study. JY, XY, CZ constructed the recombinant GS115-pW317-*MCPD*. LH, HD, LL and ZY contributed significantly to analysis and article preparation. WY performed the data analyses. XP helped evaluate the immune efficacy. All authors contributed to the article and approved the submitted version.

## Funding

The research was supported by the National Key R&D Program of China (2019YFD0900104), the key project program of Huzhou City (2021GZ28, 2021GZ24, 2021GZ31), and “San Nong Liu Fang” Science and Technology Collaboration Projects (2020SNLF020).

## Conflict of Interest

The authors declare that the research was conducted in the absence of any commercial or financial relationships that could be construed as a potential conflict of interest.

## Publisher’s Note

All claims expressed in this article are solely those of the authors and do not necessarily represent those of their affiliated organizations, or those of the publisher, the editors and the reviewers. Any product that may be evaluated in this article, or claim that may be made by its manufacturer, is not guaranteed or endorsed by the publisher.
