# Supplementary material for: Oral Vaccination With Recombinant Pichia pastoris Expressing Iridovirus Major Capsid Protein Elicits Protective Immunity in Largemouth Bass (Micropterus salmoides)
Source: Front Immunol. 2022 Mar 4;13:852300. doi: 10.3389/fimmu.2022.852300 (PMC8931665; doi:10.3389/fimmu.2022.852300)
Supplement: Supplementary file 1 [file DataSheet_1.pdf]

Sequence information of MCPD protein

1. Amino acid sequence MCP protein

MSSVTGSGITSGFIDLATYDSLDKALYGGKDATTYFVKEHYPVGWFTKLPTAATKTSGTPAFGQHFSVGVP
RSGDYVLNSWLVLKTPQIKLLAANQFNNDGTIRWTKNLMHNVVEHAALSFNEIQAQQFNTAFLDAWNEY
TMPEAKRIGYYNMIGNTSDLVNPAPATGQAGARVLPAKNLVLPLPFFFGRDSGLALPTVTLPYNEIRITISLR
SIQDLLILQHKTTGEVKPIVATDLEGGLPDTVEAHVYMTVGLVTAAERQAMSSSVRDMVVEQMQMAPVH
MVNPKNATVFHADLRFSHAVKALMFMVQNVTHKSVGSNYTCVTPVVGAGNTVLEPALAVDPVKSASLV
YENTTRLPDMSVEYYSLVQPWYYAPAIPISTGHHLYSYALSLNDPHPSGSTNFGRLTNASINVSLSAEAGTA
AGGGGADNSGYKNPQKYALVVMAINHNIIRIMNGSMGFPILHHHHHH.

Sequence marked red was major epitopes of MCPD

2. Nucleotide sequence corresponding to major epitopes of MCPD

GGTACCATCAGATGGACCAAAAATCTCATGCACAACGTTGTGGAGCACGCCGCACTCT
CGTTCAACGAGATTCAGGCCCAGCAGTTTAACACTGCTTTCCTGGACGCCTGGAACGA
GTACACCATGCCCGAGGCCAAGCGCATCGGCTACTACAACATGATTGGCAACACTAGC
GATCTCGTCAATCCCGCCCCCGCCACCGGTCAAGCAGGAGCTAGGGTCCTGCCCGCCA
AAAACCTTGTCCTTCCTCTCCCCTTCTTTTTCGGCAGAGACAGCGGGCTGGCCCTGCCT
ACAGTCACCCTGCCTTACAACGAAATTAGAATCACCATCAGCCTGAGATCCATTCAGGA
TCTCCTGATTCTTCAGCACAAGACGACCGGAGAAGTCAAGCCCATCGTGGCCACAGAT
CTGGAAGGAGGTCTCCCAGACACGGTAGAGGCTCACGTCTACATGACTGTGGGTCTGG
TGACTGCCGCCGAGCGTCAGGCTATGAGCAGCTCAGTCAGGGACATGGTGGTGGAGC
AGATGCAGATGGCTCCGGTCCACATGGTCAACCCCAAGAACGCCACCGTCTTTCACGC
AGACCTGCGCTTTTCCCACGCCGTCAAAGCGCTCATGTTTATGGTGCAAAACGTCACT
CACAAGTCTGTGGGTTCCAACTACACTTGCGTCACTCCTGTTGTTGGAGCGGGTAACA
CCGTCCTGGAGCCCGCCCTGGCCGTCGATCCGGTCAAGAGCGCCAGTCTGGTGTACGA
AAACCACCACCAC CACCACCACTAA
